# Distinct Sleep Alterations in Alcohol Use Disorder Patients with and without Korsakoff’s Syndrome: Relationship with Episodic Memory

**DOI:** 10.3390/jcm12062440

**Published:** 2023-03-22

**Authors:** Alice Laniepce, Shailendra Segobin, Claire André, Françoise Bertran, Céline Boudehent, Najlaa Lahbairi, Angéline Maillard, Alison Mary, Laurent Urso, François Vabret, Nicolas Cabé, Anne-Lise Pitel, Géraldine Rauchs

**Affiliations:** 1 Normandie Univ, UNICAEN, PSL Université, EPHE, INSERM, U1077, CHU de Caen, GIP Cyceron, NIMH, 14000 Caen, France; 2 Normandie Univ, UNIROUEN, CRFDP (EA 7475), 76000 Rouen, France; 3 Normandie Univ, UNICAEN, INSERM, U1237, PhIND “Physiopathology and Imaging of Neurological Disorders”, Team NeuroPresage, Institut Blood and Brain @ Caen-Normandie, Cyceron, 14074 Caen, France; 4 Unité D’exploration et de Traitement des Troubles du Sommeil, CHU de Caen, 14000 Caen, France; 5 Addiction Department, CHU de Caen, 14000 Caen, France; 6 Addiction Department, Centre Hospitalier de Roubaix, 59100 Roubaix, France; 7 Institut Universitaire de France (IUF), 75231 Paris, France

**Keywords:** Korsakoff’s syndrome, alcohol use disorder, polysomnography, sleep, episodic memory

## Abstract

Alcohol Use Disorder (AUD) results in sleep disturbances that may have deleterious impacts on cognition, especially on memory. However, little is known about the sleep architecture in patients with Korsakoff’s syndrome (KS). This study aims at characterizing sleep disturbances in KS compared to AUD without KS and at specifying the relationships with cognitive impairments. Twenty-nine AUD patients (22 without KS and 7 with KS) and 15 healthy controls underwent a neuropsychological assessment and a polysomnography. The severity of sleep-disordered breathing and sleep fragmentation was similar in AUD and KS patients compared to controls. Sleep architecture differed between both patient groups: the proportion of slow-wave sleep was reduced in AUD patients only, while a lower proportion of rapid-eye movement (REM) sleep was specifically observed in KS patients. The proportion of REM sleep correlated with the severity of episodic memory deficits when AUD and KS were examined together. These data provide evidence for both similarities and specificities regarding sleep alterations in AUD patients with and without KS. They also indicate that altered sleep architecture may contribute to the pathophysiology of alcohol-related memory disorders.

## 1. Introduction

Korsakoff’s syndrome (KS), a neurological complication most frequently induced by the combination of chronic and heavy alcohol consumption and thiamine deficiency [1], is characterized by a disproportionate impairment of episodic memory compared to other cognitive deficits such as executive dysfunction [2,3]. Up to 50% of Alcohol Use Disorder (AUD) patients without KS also present neuropsychological deficits, notably affecting episodic memory and executive functions. The severity of these deficits ranges from mild to moderate in most cases and can become severe in some patients at risk for KS [4,5,6]. In addition, AUD patients present sleep disturbances, including difficulties falling asleep, sleep fragmentation (notably increased time spent awake after sleep onset), lower proportions of slow wave sleep (SWS), and higher proportions of rapid eye movement (REM) sleep compared to age-matched healthy controls [7]. Sleep-disordered breathing is also common in AUD [8]. These sleep disturbances can persist for several months after alcohol withdrawal [9] and contribute to memory alterations [10].

Studies investigating sleep architecture in KS patients are scarce and provide mixed results. Regarding sleep complaints, we reported that they were less frequent in KS patients compared to AUD without KS and inversely correlated with the severity of executive deficits and structural brain alterations, suggesting an impaired ability to self-evaluate sleep quality in KS patients rather than the presence of good sleep quality [11]. Regarding sleep architecture, studies showed divergent results. Some studies reported REM sleep abnormalities [12,13] and higher sleep fragmentation compared to healthy controls or patients with Alzheimer’s disease [12,14], whereas one study reported no difference compared to controls [15]. Moreover, several limitations can be highlighted concerning the inclusion criteria for KS patients, the method used to define the sleep stages, and the absence of a control group. To our knowledge, no study has directly compared objective sleep quality between AUD and KS patients. Moreover, the relationship between sleep quality and cognitive performance has never been explored in KS. A better understanding of sleep architecture alterations in KS patients will be particularly relevant for clinical care since sleep disturbances are an important issue in the care of KS patients. KS patients are often cared for in institutionalized arrangements for years, and sleep problems may remain undetected because of their severe neurocognitive deficits [16] and the reduction of diurnal activity [17]. However, sleep problems may exacerbate psycho-affective symptoms such as irritability and depression [18] and contribute to cognitive deficits, including executive [19] and memory deficits [19].

The present study aimed at determining whether (1) some sleep parameters are specifically impaired in KS patients compared with AUD patients and whether (2) they relate to the severity of cognitive deficits.

## 2. Materials and Methods

### 2.1. Participants

Twenty-two AUD patients (all men), seven KS patients (all women), and 15 healthy controls (HC; 13 men) were included in the present study. None of them had a history of neurological or endocrine or other infectious diseases, previously diagnosed sleep disorders (sleep-disordered breathing, primary insomnia, periodic limb movement disorder, etc.), depression (assessed using both the Beck Depression Inventory (BDI) [20] and a psychiatric assessment), or other forms of substance use disorder (except tobacco). All participants completed questionnaires related to anxiety states (STAI-A) and anxiety traits (STAI-B) with the help of a psychologist who was ready to intervene where necessary [21] (Table 1). During the study, AUD patients and HC were free from any psychotropic medication, while some KS patients were taking medications, including psychotropic drugs prescribed many years ago, which reflects the clinical reality of KS (Table 2).

All participants were informed about the studies approved by the local ethics committee (for the Alcobrain study: CPP Nord-Ouest III, n°. IDRCB: 2011-A00495-36; for the Age-Well study: CPP Nord-Ouest III, n° IDRCB: 2016-A01767-44; EudraCT: 2016-002441-36; ClinicalTrials.gov Identifier: NCT02977819) prior to their inclusion and provided their written informed consent. The Age-Well study was sponsored by the French National Institute of Health and Medical Research (Inserm).

KS patients met the DSM-5 criteria for “alcohol-induced major neurocognitive disorder, amnestic-confabulatory type, persistent” [22]. They were recruited in a nursing home dedicated to KS patients (Maison Vauban, Roubaix, France). The “Maison Vauban” is a nursing home dedicated to providing care to female patients with KS. We first met the institutional team (physician and psychologist) to set up a diagnostic commission. Each patient was included in the study after a careful selection procedure involving experts in cognitive neuropsychology and behavioral neurology. During this commission, we individually examined the case of each patient to ensure that she met the inclusion criteria: age between 18 and 70 years; at least 8 years of schooling (can read and write); native French speaker; Mini Mental State Examination (MMSE; [23]) > 20; DSM-5 criteria for “alcohol-induced major neurocognitive disorder, amnestic-confabulatory type, persistent”; a history of long-term chronic and excessive drinking (this criteria was confirmed by the family and/or medical records). Clinical and neuroimaging examinations enabled us to ensure that KS patients showed a stable clinical and cognitive profile (severe and persistent anterograde amnesia without recent deterioration) and to exclude other possible etiologies explaining memory disorders (including focal brain damage or neurodegenerative diseases). All KS patients had a history of heavy drinking, but their amnesia made it difficult to obtain accurate information about their alcohol consumption. The background information collected for the patients with KS came mainly from family members and medical records. A detailed neuropsychological examination enabled the diagnosis of all KS patients who presented severe episodic memory deficits potentially associated with executive dysfunctions. All patients had been diagnosed several years ago; most of them have been living in the nursing home for more than ten years, and all of them presented a stable clinical and cognitive profile (see [24] for a description of the pattern of cognitive performance, brain shrinkage, and metabolic alterations).

AUD patients were recruited by clinicians among inpatients in the Addiction Department at Caen University Hospital. Although the patients were early in abstinence (10.81 ± 3.80 days of sobriety prior to inclusion), none of them presented physical symptoms of alcohol withdrawal as assessed by the Cushman’s scale at inclusion [25]. They were interviewed based on a modified version of the semi-structured lifetime drinking history [26] and the Alcohol Use Disorders Identification Test (AUDIT, [27]). The measures included the duration of AUD (in years), the number of previous alcohol withdrawals, and daily alcohol consumption prior to treatment (in units; a standard drink corresponding to a beverage containing 10 g of pure ethanol).

HC were recruited in such a way as to match the demographics (age, gender, and education) of AUD patients. Among the 15 HC, 3 were enrolled in the Age-Well clinical trial [28]. The baseline data of these participants was analysed. HC were screened with the AUDIT questionnaire to ensure that they did not meet the criteria for alcohol abuse (AUDIT < 7 for men and <6 for women). None of the controls had a BDI score > 29, a Mattis Dementia Rating score < 129 [29], complained about their sleep (Pittsburg Sleep Quality Index (PSQI) ≤ 5; [30]), nor presented signs of daytime sleepiness as assessed by the Epworth Sleepiness Scale (ESS < 8; [31]).

### 2.2. Sleep Recording

All participants underwent a polysomnography (PSG) examination using an ambulatory recording device (Siesta^®^, Compumedics, Abbotsford, Australia), allowing AUD patients to sleep in the addiction department, KS in the nursing home, and HC at home. They were instructed to follow their habitual sleep-wake schedule. No participant had a previous diagnosis of a sleep disorder; PSG were thus performed without CPAP therapy for sleep disordered breathing. The PSG recording was conducted the same week as the neuropsychological examination and included 20 electroencephalography (EEG) electrodes (Fp1, Fp2, F3, F4, F7, F8, T3, T4, C3, C4, P3, P4, O1, O2, FZ, CZ, PZ, vertex ground, and a bi-mastoid reference) placed over the scalp according to the international 10–20 system, with impedance kept below 5 kΩ. We also recorded the electrooculogram (EOG), chin electromyogram (EMG), electrocardiogram (ECG), respiratory movements using abdominal and thoracic belts, respiratory airflow using nasal and oral thermistors, and oxygen saturation with a finger pulse oximeter. The EEG signal was digitalized at a sampling rate of 256 Hz. High-pass and low-pass filters were applied, respectively, at 0.3 Hz and 35 Hz. PSG recordings were scored in 30-s epochs according to the American Association of Sleep Medicine standard criteria [32].

For each PSG recording, the following parameters were obtained: total sleep time (TST; in minutes), sleep efficiency (SE (%), corresponding to the ratio between time spent asleep and time in bed), sleep onset latency (in minutes, referring to the time from lights-off to the first three epochs of any stage of sleep), wake after sleep onset (WASO; in minutes), arousal index (number of arousals/TST), stage shift index (number of sleep stage transitions to N1/TST), the Apnea-Hypopnea Index (AHI, corresponding to the number of respiratory events per hour of sleep), and the proportion of TST spent in each sleep stage (N1, N2, N3, and REM sleep).

All participants underwent a self-assessment of their daytime sleepiness pattern using the Epworth Sleepiness Scale (ESS) [31] and sleep quality using the PSQI [30]. The ESS is a self-assessment questionnaire consisting of eight items. The participants were asked to rate, on a four-point scale (0–3), their usual chances of dozing off or falling asleep while engaged in eight different activities. The total score ranges from 0 to 24. The higher the ESS score, the higher that person’s average sleep propensity in daily life, or their “daytime sleepiness”. The PSQI is a 19-item self-assessment questionnaire that allows to assess sleep quality and sleep disturbances over the last month. Seven items are evaluated, ranging from 0 to 3: subjective sleep quality, sleep latency, sleep duration, habitual sleep efficiency, sleep disturbances, use of sleeping medication, and daytime dysfunction. The total score ranges from 0 (indicating no difficulty) to 21 (major sleep difficulties). As recommended, a cutoff score of 5 was used to indicate a significant sleep complaint. The PSQI was performed in its original version (assessing sleep quality and disturbances over the past month) for HC and KS, and with an adapted version (focusing on the previous week to better reflect the different stages of alcohol treatment) for AUD patients [33]. This version was developed with the authors’ permission.

### 2.3. Neuropsychological Assessment

All participants underwent a detailed neuropsychological examination, and z-scores were systematically calculated using the means and standard deviations of control participants.

Processing speed was assessed with the Trail Making Test (TMT) [34] and the Stroop test [35]. We used the time in seconds needed to complete TMT-part A and the name condition of the Stroop test. As these two measures correlated with each other in the entire group of participants, we computed a processing speed composite z-score (the average of the two z-scores). For the assessment of the executive functions, we used three tests assessing manipulation of information (verbal backward span of the WAIS-III; [36]), inhibition (Stroop Test; time in seconds needed to complete the interference condition minus time needed for the naming condition), and flexibility (TMT; time in seconds needed to complete part B minus time needed for part A). As the three measures correlated with each other within the entire group of participants, we computed an executive composite z-score (the average of the three z-scores) to limit the number of comparisons. Short-term memory was assessed with the forward verbal span task of the WAIS-III [36]. Episodic memory was examined with the delayed free recall of the French version of the Free and Cued Selective Reminding Test (FSCRT; [37]).

### 2.4. Statistical Analyses

To test the differences between HC, AUD, and KS patients, non-parametric Kruskall-Wallis ANOVAs were conducted on demographic variables (age and education), cognitive z-scores, and sleep parameters. When appropriate, *post-hoc* comparisons were performed using Mann-Whitney tests. Then, the relationships between cognitive measures and sleep parameters that were significantly more altered in KS patients than in AUD patients were examined in the whole group of patients (AUD and KS pooled together) with Spearman correlations. The statistical threshold was set to *p* < 0.05. Effect sizes were calculated for Kruskall-Wallis ANOVAs [using the epsilon square (*ε*^2^) interpreted as 0.01 < *ε*^2^ < 0.04 (small effect), 0.04 < *ε*^2^ < 0.16 (moderate effect), and *ε*^2^ > 0.16 (large effect)] and for Mann-Whitney tests [using the rank biserial correlation (*r*) interpreted as *r* < 0.3 (small effect), 0.3 < *r* < 0.5 (moderate effect) and *r* > 0.5 (large effect); [38].

## 3. Results

### 3.1. Between-Group Comparisons on Demographic and Clinical Variables

Kruskall-Wallis ANOVAs (HC vs. AUD vs. KS) did not reveal any significant group effect for age [H_(2, N = 44)_ = 5.23, *p* = 0.07, *ε*^2^ = 0.12], education [H_(2, N = 44)_ = 3.78, *p* = 0.15, *ε*^2^ = 0.08] and body mass index [H_(2, N = 43)_ = 1.63, *p* = 0.44, *ε*^2^ = 0.04]. A Chi^2^-test showed a difference in the sex ratio between groups (χ^2^ = 27.2, *p* < 0.001). The gender-based repartition was significantly different between HC and AUD (χ^2^ = 8.48, *p* < 0.004), HC and KS (χ^2^ = 8.56, *p* < 0.003), and AUD and KS (χ^2^ = 29, *p* < 0.001). There was a significant group effect at the depression [H_(2, N = 41)_ = 15.23, *p* < 0.001, *ε*^2^ = 0.38], and anxiety-trait levels [H_(2, N = 43)_ = 12.83, *p* = 0.002, *ε*^2^ = 0.30], but none was observed at the anxiety-state level [H_(2, N = 43)_ = 5.05, *p* = 0.08, *ε*^2^ = 0.12]. Compared to HC, AUD patients were more depressed [U = 27, *p* < 0.001, *r* = 0.80] and exhibited higher levels of anxiety-traits [U = 51, *p* < 0.001, *r* = 0.70]. No difference was observed between HC and KS patients or between AUD and KS patients (*p* > 0.05) regarding depression and anxiety traits. As expected, AUD patients presented higher AUDIT scores than HC [U = 0, *p* < 0.001, *r* = 1]. Demographic and alcohol-related variables are presented in Table 1.

### 3.2. Between-Group Comparisons on Sleep Measures

**Figure 1:**
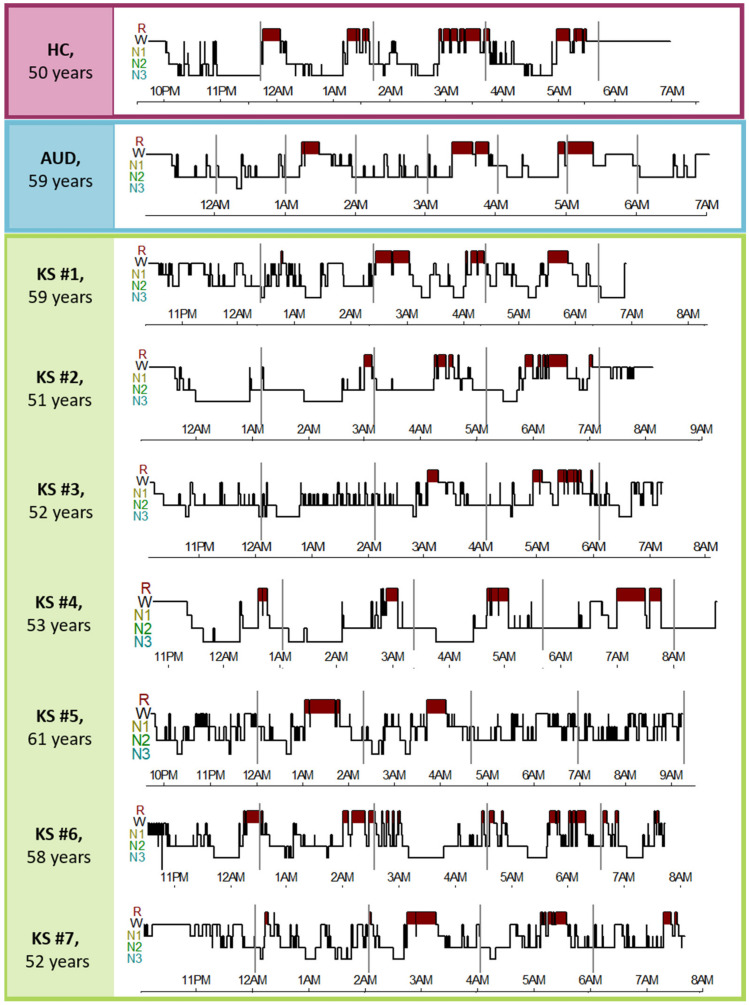
Hypnogram of each KS patient was compared to representative data obtained from one AUD patient and one HC. HC: Healthy Controls; AUD: patient with Alcohol Use Disorder; KS: patient with Korsakoff’s Syndrome.

Figure 1 depicts individual hypnograms for each KS patient and a representative example of a participant for the AUD and HC groups.

There was no significant group effect on sleep latency [H_(2, N = 44)_ = 5.11, *p* = 0.07, *ε*^2^ = 0.11], sleep efficiency [H_(2, N = 44)_ = 0.57, *p* = 0.75, *ε*^2^ = 0.01], WASO [H_(2, N = 44)_ = 1.77, *p* = 0.41, *ε*^2^ = 0.04], and the proportion of N2 sleep [H_(2, N = 44)_ = 3.52, *p* = 0.17, *ε*^2^ = 0.08]. Kruskall-Wallis tests revealed a significant group effect on sleep duration [H_(2, N = 44)_ = 9.78, *p* = 0.008, *ε*^2^ = 0.23], stage shifts index [H_(2, N = 44)_ = 6.26, *p* = 0.01, ε^2^ = 0.30] and AHI [H_(2, N = 44)_ = 15.80, *p* < 0.001, *ε*^2^ = 0.37]. KS patients slept longer than AUD patients (U = 18, *p* = 0.002, *r* = 0.77), and HC (U = 15, *p* = 0.007, *r* = 0.71), who did not differ from one another (*p* > 0.05). Compared to HC, AUD patients and KS patients presented a higher stage shifts index (U = 66, *p* = 0.002, *r* = 0.6, and U = 17, *p* = 0.01, *r* = 0.68, respectively), but did not differ from each other (*p* > 0.05). Compared to HC, AUD patients and KS patients presented a higher AHI (U = 38, *p* < 0.0001, *r* = 0.77, and U = 10, *p* = 0.02, *r* = 0.63, respectively), but did not differ from each other (*p* > 0.05).

**Figure 2:**
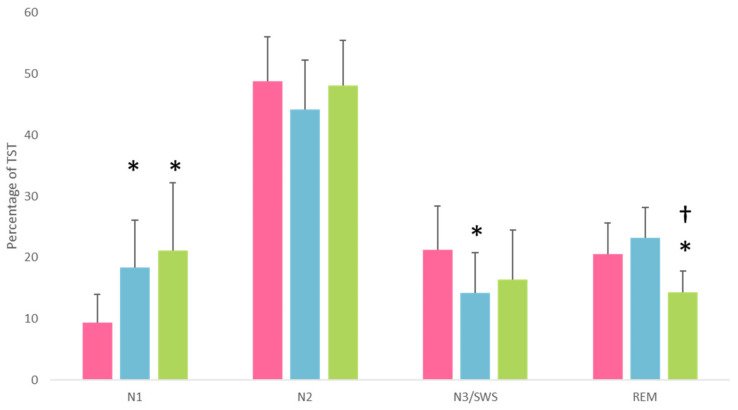
Time spent in each sleep stage is expressed as a percentage of total sleep time according to the group (HC vs. AUD vs. KS). *: significant difference compared to HC; †: significant difference compared to AUD. HC, AUD, and KS patients were represented in pink, blue, and green, respectively. TST: total sleep time.

We also found a significant group effect on the proportion of N1 sleep [H_(2, N = 44)_ = 16.36, *p* < 0.001, *ε*^2^ = 0.38], N3 sleep [H_(2, N = 44)_ = 6.86, *p* = 0.03, *ε*^2^ = 0.16], and REM sleep [H_(2, N = 44)_ = 12.51, *p* = 0.002, *ε*^2^ = 0.30]. Compared to HC, AUD patients and KS patients had a higher proportion of N1 sleep (U = 42.5, *p* < 0.001, *r* = 0.74, and U = 13, *p* = 0.006, *r* = 0.75, respectively), but did not differ from each other (*p* > 0.05). Compared to HC, AUD patients presented a lower proportion of N3 sleep (U = 77, *p* = 0.007, *r* = 0.53), while the proportion of this sleep stage did not differ between HC and KS patients (*p* > 0.05). KS patients had a lower proportion of REM sleep compared to AUD patients and HC (U = 12, *p* = 0.001, *r* = 0.84, and U = 17.5, *p* = 0.01, *r* = 0.67, respectively), who did not differ from each other (*p* > 0.05). The differences in the proportions of the different sleep stages are represented in Figure 2.

Given the high prevalence of sleep-disordered breathing in AUD (82% of patients) and KS (71% of patients), we performed complementary analyses using ANCOVAs with the AHI as a covariate. The results reported above remained significant, except for the main group effect on N1 and N3 sleep (see Table A1 for details). In addition, given that two KS patients were taking antidepressant drugs (duloxetine and venlafaxine; Table 2) during the study, we performed a second complementary analysis with the quantity of antidepressant (in milligrams) used as another covariate. The results remained unchanged (see Table A2 for details).

There was no significant group effect (HC vs. AUD vs. KS) on the ESS total score [H_(2, N = 44)_ = 0.14, *p* = 0.93, *ε*^2^ = 0.003]. A significant group effect was observed on the PSQI total score [H_(2, N = 42)_ = 26.6, *p* < 0.001, *ε*^2^ = 0.65] with a graded effect. AUD patients achieved a higher PSQI score than KS patients (U = 21, *p* = 0.007, *r* = 0.7), who complained more about their sleep compared to HC (U = 15, *p* = 0.008, *r* = 0.7). Regarding the PSQI components, a significant group effect was observed on subjective sleep quality [component n°1; H_(2, N = 42)_ = 17.85, *p* < 0.001, *ε*^2^ = 0.043], sleep latency [component n°2; H_(2, N = 42)_ = 11.47, *p* = 0.033, *ε*^2^ = 0.28], sleep duration [component n°3; H_(2, N = 42)_ = 17.21, *p* < 0.001, *ε*^2^ = 0.42], sleep efficiency [component n°4; H_(2, N = 44)_ = 12.45, *p* = 0.002, *ε*^2^ = 0.3] and daytime dysfunction [component n°7; H_(2, N = 42)_ = 10.17, *p* = 0.006, *ε*^2^ = 0.25]. The other components displayed no group effect (HC vs. AUD vs. KS). *Post-hoc* comparisons showed that compared to HC, AUD patients reported poorer sleep quality [component n°1; U = 40, *p* < 0.001, *r* = 0.7], longer sleep latency [component n°2; U = 54.5, *p* < 0.001, *r* = 0.6], shorter sleep duration [component n°3; U = 50, *p* < 0.001, *r* = 0.7], lower sleep efficiency [component n°4; U = 58, *p* < 0.001, *r* = 0.6] and greater daytime dysfunction [component n°7; U = 65, *p* = 0.002, *r* = 0.6]. Compared to KS, AUD patients reported poorer sleep quality [U = 29.5, *p* = 0.01, *r* = 0.6] and shorter sleep duration [U = 19, *p* = 0.003, *r* = 0.7]. No other significant differences were observed (Table 3).

### 3.3. Between-Group Comparisons on Cognitive Performance

Kruskall-Wallis ANOVAs revealed a significant group effect on processing speed [H_(2, N = 44)_ = 11.95, *p* = 0.002, *ε*^2^ = 0.28], executive functions [H_(2, N = 44)_ = 13.93, *p* < 0.001, *ε*^2^ = 0.32], and episodic memory [H_(2, N = 43)_ = 22, *p* < 0.0001, *ε*^2^ = 0.52]. No difference was observed for short-term memory [H_(2, N = 44)_ = 3.89, *p* = 0.14, *ε*^2^ = 0.09]. *Post-hoc* comparisons showed that compared to HC, AUD patients exhibited lower performance for processing speed (U = 64, *p* = 0.002, *r* = 0.61) and executive functions (U = 63, *p* = 0.002, *r* = 0.62). Compared to HC, KS patients presented lower performance for processing speed (U = 15, *p* = 0.009, *r* = 0.71), and executive functions (U = 11, *p* = 0.004, *r* = 0.79). Concerning episodic memory, a graded effect was observed: KS patients showed lower delayed recall performance than AUD patients (U = 11.5, *p* = 0.001, *r* = 0.84), the latter also being impaired compared to HC (U = 57, *p* = 0.001, *r* = 0.97; Table 1).

### 3.4. Relationships between Episodic Memory Performance and REM Sleep Parameters in the Entire Group of Patients

**Figure 3:**
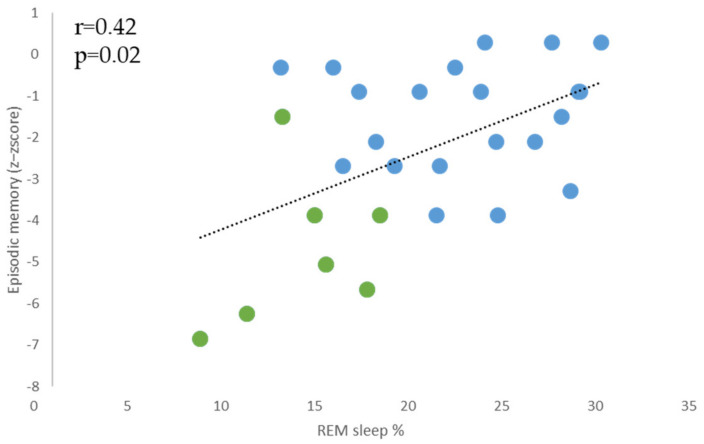
Relationships between the proportion of REM sleep and episodic memory performance when AUD and KS patients are pooled together. AUD: patients with Alcohol Use Disorder; KS: patients with Korsakoff’s Syndrome. AUD patients are represented by blue dots, and KS patients are represented by green dots.

As REM sleep and episodic memory were significantly more affected in KS than in AUD patients, we explored the relationship between the proportion of this sleep stage and episodic memory performance. In both AUD and KS patient groups pooled together, episodic memory performance was positively correlated with the proportion of REM sleep (*r* = 0.42, *p* = 0.02; Figure 3). These results remained significant when controlling for the AHI (*r* = 0.52, *p* = 0.006).

Exploratory analyses including other cognitive measures revealed that only the episodic memory score was correlated with the proportion of REM sleep (data not shown).

## 4. Discussion

The present study aimed at determining whether (1) some sleep parameters are specifically impaired in KS patients compared with AUD patients and (2) they relate to the severity of cognitive deficits. We found that both AUD and KS patients presented with sleep-disordered breathing and sleep fragmentation (i.e., an increase in stage shifts). In addition, we observed both similarities and specificities regarding the alterations of sleep architecture in AUD patients with or without KS.

Our study confirms sleep fragmentation in AUD and KS patients [7,14] and indicates that it may be explained by sleep-disordered breathing. In the present study, based on the criteria set forth in the third edition of the International Classification of Sleep Disorders [39], we showed that 82% of AUD patients and 71% of KS patients presented moderate to severe sleep-disordered breathing (AHI ≥ 15). This prevalence of sleep-disordered breathing in AUD and KS is higher than in the general population, as epidemiological studies reported sleep disordered breathing (SDB) in 24% of men and 9% of women [40]. In the present study, the gender ratio in the AUD and KS groups rendered it impossible to investigate a potential gender bias in the prevalence of sleep-disordered breathing in the patient groups. Among the few studies that investigated sleep breathing disorders in AUD patients, one reported no effect of gender on sleep breathing disorders [41]. The high frequency observed in the present study may result from a relaxation of the dilator muscles of the upper airways due to chronic and excessive alcohol consumption [8]. Sleep-disordered breathing is observed in AUD and KS patients even in the absence of a high BMI or clinical symptoms such as excessive daytime sleepiness. This absence of excessive daytime sleepiness may be explained by the clinical presentation of SDB, which differs depending on gender [42]. Indeed, men more frequently present snoring, gasping, and apneas, whereas women tend to present daytime fatigue, nightmares, morning headaches, and mood disturbances [42,43]. As a consequence, the screening tools for sleep apnea are less sensitive when it comes to identifying women with SDB, especially when the disease is mild [42]. This was confirmed by the data from the Sleep Heart Health study, which showed that although women were as likely as men to report daytime sleepiness, they were less likely to obtain an abnormal score on the Epworth Sleepiness Scale (ESS) [42]. In the present study, no differences were observed in the ESS total score between groups (HC vs. AUD vs. KS), despite the high prevalence of SDB in patients (AUD and KS). In the KS group, composed only of women, this result can be interpreted in two ways. First, severe cognitive deficits may alter the ability to self-evaluate the consequences of disturbed sleep on daytime functioning. Second, the ESS may not be sensitive enough to target diurnal symptoms in women [42]. This last hypothesis cannot be verified since we did not collect information about the presence of fatigue, nightmares, and morning headaches. Sleep-disordered breathing has been associated with negative consequences such as brain damage [44], cognitive deficits [45], an increased risk of cardiovascular diseases [46], or, more generally, a higher risk of mortality [47]. These findings highlight the clinical relevance of exploring these sleep disturbances in AUD patients with and without KS by means of sleep questionnaires, medical staff observation, and objective measures when appropriate.

Besides these sleep disturbances commonly reported in AUD and KS patients, the novelty of the present study relies on the evidence of specific sleep alterations depending on the clinical form (AUD or KS). First, the presence of a sleep complaint [11] and the lower proportion of slow-wave sleep (N3) observed in AUD patients are in agreement with previous studies conducted in recently detoxified patients [7,48]. Slow-wave sleep alterations could be related to the direct effects of chronic and excessive alcohol consumption and/or withdrawal severity [33,49]. In contrast, the relative preservation of slow-wave sleep in KS patients may be explained by a recovery of sobriety in some brain regions involved in this sleep stage. The frontal cortex is known to contribute to the generation of slow waves [50] and has recently been found to show some recovery with prolonged abstinence in this same sample of KS patients [24]. A normalization of the proportion of slow-wave sleep has already been reported in long-term abstinent AUD patients [51,52].

Our study also highlights that KS patients complain less about their sleep than AUD patients. In a previous study [11], conducted in a larger group of KS patients, some of whom are also included in the present work, we found that these less frequent sleep complaints were associated with more severe executive deficits and structural brain abnormalities. As severe cognitive deficits in KS patients may alter their ability to self-assess [16], the lack of group difference in the ESS total score does not necessarily indicate an absence of sleepiness. However, the use of a sleep questionnaire in clinical practice should not be abandoned since it reflects the subjective perception of the patients’ mental states and feelings [11]. Another explanation could be that KS patients, who are at a chronic and stable stage of the disease, benefit from living in a serene and sober environment, stay away from alcohol, and therefore sleep more and complain less about their sleep.

Regarding sleep architecture, KS patients were historically investigated to better understand the relationship between sleep and dreams in amnestic patients. First, Lairie et al. (1966) reported a higher proportion of REM sleep in three KS patients who had been diagnosed for less than two years [12]. Greenberg et al. (1968) found that the proportion of REM sleep was related to the duration of KS [13]. More precisely, the authors reported that three recently diagnosed KS patients (i.e., less than one year) had a “high dream percentage”, while three KS patients who had been diagnosed for more than one year showed normal or “low dream times” [13]. Assal et al. (1970) reported that “phases with eye movements are rare and morcelled” in KS patients (Assal 1970 cited in [12]). Two other studies reported higher sleep fragmentation in KS patients compared to healthy controls [12] or patients with Alzheimer’s disease [14]. Cathala et al. (1988) reported no difference in sleep architecture in 15 KS patients (diagnosed for at least 1 year) compared to 15 healthy controls [15]. In the present study, using a validated method to define sleep stages and a refined inclusion procedure for KS patients, we observed an alteration of REM sleep, which is in agreement with two previous studies ([12], Assal et al. (1970) cited in [13]). Such an alteration of REM sleep has already been documented in other diseases such as Mild Cognitive Impairment [53] and Alzheimer’s disease [54] and is related to cognitive decline [55,56] and an increased risk of dementia [57].

The origin of sleep disturbances in KS patients is thought to be multifactorial. First, REM sleep alterations observed in KS patients may result from the effect of thiamine deficiency or altered thiamine metabolism. Indeed, thiamine deficiency has been associated with a smaller proportion of REM sleep in rats [58] and with sleep disturbances in humans [59]. Moreover, the cholinergic system is known to play a crucial role in REM sleep generation [60] and memory [61,62]. Interestingly, studies in rats have demonstrated that thiamine deficiency decreases the number of cholinergic neurons in the basal forebrain [63], resulting in dysfunction of the cholinergic system [64]. Basal forebrain alterations have been reported in KS patients [61]. We can thus hypothesize that a lower proportion of REM sleep in KS patients may result from irreversible brain damage in the cholinergic system, potentially due to thiamine deficiency or altered thiamine metabolism. This hypothesis is also supported by the relationship observed between a lower proportion of REM sleep and episodic memory deficits in AUD and KS patients. REM sleep alterations may thus contribute to the cognitive pathophysiology of amnesia in KS and potentially beyond. This hypothesis should be considered with caution due to the absence of thiamine measurements in the present study. Further studies are needed to assess the relationships between thiamine deficiency and sleep alterations in AUD with and without KS and the implication of the cholinergic system. Second, the REM sleep alterations observed in the present study may result from the side effects of psychotropic medications. A few KS patients had been taking psychotropic drugs for many years (about 10 years) at the moment of inclusion. One could argue that these drugs may contribute to the observed sleep alterations. We conducted additional analyses (Table A3) using the quantification of benzodiazepines as a covariate (converted to the equivalent dose of diazepam in mg). We found that KS patients still presented a lower proportion of REM sleep than AUD patients but did not differ any more from controls. This finding is in accordance with the literature [65], which indicates a reduction in time spent in REM sleep associated with the use of benzodiazepines. However, most KS patients receive psychotropic medications in their usual living conditions, which implies that the present findings reflect the clinical reality of KS patients [66,67]. Second, given that the prescription had remained unchanged for many years, it is difficult to determine whether the effect of psychotropic medication on sleep remains significant [66].

The main strength of the present study is its exploration of sleep architecture in AUD and KS patients, carefully recruited according to stringent criteria. These patients underwent a detailed neuropsychological assessment performed close in time to the sleep recording (during the same week). The number of KS patients in this study is relatively limited, but similar to the number of KS patients included in previously published studies [14,24,68,69]. It is rare and difficult to perform sleep recordings in patients with long-term amnesia living in an institution. However, some limitations must be mentioned. First, we observed that KS patients slept more than AUD and controls. This finding may result from our methodological design, notably because the night of PSG was not performed under the same experimental conditions for all groups, which is a limitation of the present study. While both AUD patients (who slept at the hospital) and HC subjects (who slept at home and had professional duties) had constraints on waking time, KS patients (who slept at the nursing home) were not limited in their sleep duration. Further studies using similar recording conditions between patients are warranted to confirm these results. Second, our study did not consider the potential gender differences. However, a narrative review suggests a gender bias for episodic memory, with women performing higher than men on verbal episodic memory tasks in healthy populations [70]. Regarding AUD and KS, only a few studies have investigated this issue. In patients with AUD, the severity of memory deficits is similar between men and women [71]. Regarding patients with KS, a recent study reported no gender effect on episodic memory performance [72]. Although we cannot assess this potential bias in our study, which constitutes a limitation, the studies reported above suggest that the observed group difference in episodic memory is unlikely to be attributed to a gender effect. Concerning a potential gender effect on sleep architecture, data collected in the general population indicate that women tend to have less time spent awake during the night, shorter sleep onset latency, better sleep efficiency, and a higher percentage of SWS compared to men [73]. The results are less clear in the AUD population, due notably to the limited number of studies investigating the interaction between AUD and gender on sleep architecture during early and long-term alcohol abstinence [7,73]. No gender differences were identified concerning the frequency of insomnia in AUD patients early in abstinence [74]. To our knowledge, only one study has examined sleep architecture in long-term abstinent AUD men and women [75]. The authors showed that both male and female alcoholics had lower delta activity during NREM sleep than sex-matched controls and that the interaction between gender and diagnosis was not significant. Given the limited preexisting literature, gender effects on sleep architecture in AUD and KS patients still need to be explored. Further studies are thus required to assess a potential gender effect on behavior, cognition, and sleep architecture in AUD and KS.

## 5. Conclusions

The present study provides evidence for both similarities and specificities regarding sleep alterations in AUD patients with and without KS. Sleep-disordered breathing was frequent in AUD and KS patients and will lead, if untreated, to sleep fragmentation with harmful consequences for cognitive performance and brain integrity. Therefore, this sleep disorder should be more systematically assessed and treated in these patients. Sleep disturbances may have consequences for emotional and cognitive functioning, such as an increase in irritability and poor attentional and memory performance. These symptoms may also be associated with negative health outcomes, including increased sedentary behavior and an unhealthy lifestyle, as well as an elevated mortality risk among these patients. We also provide evidence of specific sleep alterations according to the clinical form: a smaller proportion of slow-wave sleep in AUD and a smaller proportion of REM sleep in KS. The proportion of REM sleep may contribute to the pathophysiology of alcohol-related memory disorders.

## Figures and Tables

**Table 1:** Demographic, clinical, neuropsychological, and alcohol-related data in HC, AUD, and KS patients.

	HC N = 15	AUD N = 22	KS N = 7	Statistics ^a^	Group Comparisons
Demographic data					
Age (years)	48.3 ± 11.3	47.6 ± 9.7	56.1 ± 4	H_(2, N = 44)_ = 5.23, *p* = 0.07	NS
Education (years)	11.8 ± 0.6	11.8 ± 2.5	10.4 ± 1.1	H_(2, N = 44)_ = 3.78, *p* = 0.15	NS
Sex ratio (W/M)	5/10	0/22	7/0	χ^2^ = 27.2, *p* < 0.001	(HC = AUD) ≠ KS
Body Mass Index (kg/m^2^)	24.3 ± 4	23.2 ± 2.7	26 ± 6.3	H_(2, N = 43)_ = 1.63, *p* = 0.44	NS
STAI-A	25.8 ± 4.9	32.3 ± 10.1	30.7 ± 12.4	H_(2, N = 43)_ = 5.05, *p* = 0.08	HC < AUD; HC = KS; AUD = KS
STAI-B	33.3 ± 7.7	47 ± 28.5	40.5 ± 10.9	H_(2, N = 43)_ = 12.83, *p* = 0.002	NS
BDI	2.6 ± 2.3	13.4 ± 9.9	8.9 ± 6.4	H_(2, N = 41)_ = 15.23, *p* < 0.001	HC < AUD; HC = KS; AUD = KS
Alcohol-related variables					
AUDIT	2 ± 1.1	28.5 ± 5	NA	U = 0, *p* < 0.001	HC < AUD
Alcohol use disorder severity (number of DSM-5 criteria)	/	9.04 ± 1.43	NA		/
Duration of alcohol use disorder (years)	/	25.63 ± 11.27	NA		/
Daily alcohol consumption before entry (units/day †)	/	18.86 ± 8.12	NA		/
Number of previous alcohol withdrawal episodes	/	1.95 ± 1.29	NA		/
Abstinence (number of days before inclusion)	/	10.81 ± 3.80	NA		/
Cognitive functions (z-score)					
Processing speed	0 ± 0.8	−1.3 ± 1.5	−1.7 ± 1.4	H_(2, N = 44)_ = 11.95, *p* = 0.002	HC > (AUD = KS)
Short-term memory	0 ± 1	−0.5 ± 1	−0.8 ± 0.8	H_(2, N = 44)_ = 3.89, *p* = 0.14	NS
Executive functions	0 ± 0.8	−1 ± 1.2	−1.4 ± 0.9	H_(2, N = 44)_ = 13.93, *p* < 0.001	HC > (AUD = KS)
Episodic memory	0 ± 1	−1.5 ± 1.3	−4.7 ± 1.8	H_(2, N = 43)_ = 22, *p* < 0.0001	HC > AUD > KS
Sleep architecture					
Sleep onset latency (min)	31.7 ± 22.5	20 ± 15.4	11.3 ± 12.5	H_(2, N = 44)_ = 5.11, *p* = 0.07	NS
Sleep efficiency %	82.3 ± 8.7	84.2 ± 8.7	85.1 ± 6	H_(2, N = 44)_ = 0.57, *p* = 0.75	NS
Total sleep time (min)	387 ± 59.7	376 ± 65.7	465 ± 40.6	H_(2, N = 44)_ = 9.78, *p* = 0.008	(HC = AUD) < KS
WASO (min)	51.9 ± 37.3	52.3 ± 39.5	69.9 ± 37.4	H_(2, N = 44)_ = 1.77, *p* = 0.41	NS
Stage shifts to N1-sleep index (nb/h)	1.61 ± 2.01	4.73 ± 2.2	5.68 ± 2.52	H_(2, N = 44)_ = 6.26, *p* = 0.01	HC < (AUD = KS)
Apnea-Hypopnea Index (AHI, nb/h)	9.4 ± 4.3	26.2 ± 14.9	29.5 ± 21.6	H_(2, N = 44)_ = 15.80, *p* < 0.001	HC < (AUD = KS)
N1 sleep (% of total sleep time)	9.4 ± 4.5	18.4 ± 7.72	21.1 ± 11	H_(2, N = 44)_ = 16.36, *p* < 0.001	HC < (AUD = KS) ‡
N2 sleep (% of total sleep time)	48.8 ± 7.3	44.2 ± 8.1	48.1 ± 7.4	H_(2, N = 44)_ = 3.52, *p* = 0.17	NS
N3 sleep (% of total sleep time)	21.2 ± 7.2	14.3 ± 6.6	16.3 ± 8.1	H_(2, N = 44)_ = 6.86, *p* = 0.03	HC > AUD; HC = KS; AUD = KS ‡
REM sleep (% of total sleep time)	20.6 ± 5	23.2 ± 4.9	14.4 ± 3.4	H_(2, N = 44)_ = 12.51, *p* = 0.002	(HC = AUD) > KS

HC: Healthy Controls; AUD: patients with Alcohol Use Disorder; KS: patients with Korsakoff’s Syndrome; STAI: State-Trait Anxiety Inventory; BDI: Beck Depression Inventory; AUDIT: Alcohol Use Disorder Identification Test; nb: number; WASO: Wake After Sleep Onset; REM: Rapid-Eye Movement Sleep; NA: data not available; /: not applicable. ^a^: group effects were tested with Kruskall-Wallis ANOVAs followed by *post-hoc* comparisons using Mann-Whitney tests when applicable. The mean ± standard deviation are reported. †: an alcohol unit = 10 g of pure ethanol. ‡: the results did not survive after controlling for the Apnea-Hypopnea Index (AHI). BDI: there was missing data for three controls, but they completed the MADRS and presented normal scores. STAI-B: missing data for one KS patient. AUDIT: missing data for three controls, but a medical interview excluded the presence of AUD. Duration of alcohol use disorder: missing data for two AUD patients. PSQI: missing data for two AUD patients. Z-scores were calculated using the mean and standard deviation of healthy controls (see the method section for details).

**Table 2:** Individual prescription in KS patients.

KS	Gender, Age	Number of Years in the Nursing Home	Major Medication (Daily Dose)
1	W, 59	>10 years	/
2	W, 51	>10 years	Zopiclone (7.5 mg/day), Alprazolam (1.5 mg/day)
3	W, 52	>10 years	Hydroxyzine (150 mg/day), Lorazepam (3 mg/day), Duloxetine (60 mg/day)
4	W, 53	>10 years	Clonazepam (0.9 mg/day)
5	W, 61	>10 years	Venlafaxine (150 mg/day), Lorazepam (1 mg/day)
6	W, 58	>10 years	/
7	W, 52	18 months	/

/: no prescription.

**Table 3:** Daytime sleepiness and subjective sleep assessment.

	HC	AUD	KS	Statistics ^a^	Group Comparisons
ESS total score	4.5 ± 2.8	4.0 ± 2.9	5.3 ± 3.4	H_(2, N = 44)_ = 0.14, *p* = 0.93	NS
PSQI total score	2.3 ± 1.4	8.1 ± 2.9	4.6 ± 2.1	H_(2, N = 42)_ = 26.6, *p* < 0.001	HC < KS < AUD
Component 1: subjective sleep quality	0.3 ± 0.5	1.3 ± 0.7	0.6 ± 0.5	H_(2, N = 42)_ = 17.85, *p* < 0.001	HC = KS < AUD
Component 2: sleep latency	0.5 ± 0.5	1.8 ± 1.1	1.1 ± 1	H_(2, N = 42)_ = 11.47, *p* = 0.033	HC < AUD; HC = KS; AUD = KS
Component 3: sleep duration	0.3 ± 0.4	1.3 ± 0.9	0.1 ± 0.4	H_(2, N = 42)_ = 17.21, *p* < 0.001	HC = KS < AUD
Component 4: habitual sleep efficiency	0.1 ± 0.3	1.2 ± 1	0.4 ± 0.8	H_(2, N = 44)_ = 12.45, *p* = 0.002	HC < AUD; HC = KS; AUD = KS
Component 5: sleep disturbances	0.7 ± 0.5	0.9 ± 0.4	0.9 ± 0.4	H_(2, N = 44)_ = 3.59, *p* = 0.16	NS
Component 6: use of sleeping medication	0 ± 0	0.4 ± 1.1	0.8 ± 1.5	H_(2, N = 44)_ = 3.97, *p* = 0.13	NS
Component 7: daytime dysfunction	0.1 ± 0.3	1 ± 0.4	0.6 ± 1.1	H_(2, N = 42)_ = 10.17, *p* = 0.006	HC < AUD; HC = KS; AUD = KS

HC: Healthy Controls; AUD: patients with Alcohol Use Disorder; KS: patients with Korsakoff’s Syndrome; ESS: Epworth Sleepiness Scale; PSQI: Pittsburg Sleep Quality Index; ^a^: group effects were tested with Kruskall-Wallis ANOVAs followed by *post-hoc* comparisons using Mann-Whitney tests when applicable. The mean ± standard deviation are reported.

## Data Availability

All data and materials used within this study will be made available, upon reasonable request, to research groups wishing to reproduce or confirm our results.

## Appendix A

**Table A1:** Group effects (HC vs. AUD vs. KS) on objective sleep parameters using ANCOVAs with the Apnea-Hypopnea Index (AHI) as a covariate.

	Statistics	*Post-Hoc* Comparisons
Sleep onset latency	F(2, 40) = 2.6, *p* = 0.08	NS
Sleep efficiency %	F(2, 40) = 0.52, *p* = 0.59	NS
Total sleep time (min)	F(2, 40) = 5.49, *p* = 0.008	(HC = AUD) < KS
WASO (min)	F(2, 40) = 0.53, *p* = 0.59	NS
Stage shifts to N1 sleep (nb/h)	F(2, 40) = 2.02, *p* = 0.15	NS
N1 sleep (% of total sleep time)	F(2, 40) = 2.02, *p* = 0.14	NS
N2 sleep (% of total sleep time)	F(2, 40) = 1.63, *p* = 0.21	NS
N3 sleep (% of total sleep time)	F(2, 40) = 1.09, *p* = 0.35	NS
REM sleep (% of total sleep time)	F(2, 40) = 9.38, *p* = 0.0005	(HC = AUD) > KS

Group effects were tested with parametric ANCOVAs with AHI as a covariate, followed by Tukey’s test corrected for unequal sample size. NS: not significant.

**Table A2:** Group effects (HC vs. AUD vs. KS) on objective sleep parameters using ANCOVAs with the quantity of antidepressant (in milligrams) as a covariate.

	Statistics	*Post-Hoc* Comparisons
Sleep onset latency	F(2, 40) = 2.59, *p* = 0.09	NS
Sleep efficiency %	F(2, 40) = 0.25, *p* = 0.77	NS
Total sleep time (min)	F(2, 40) = 3.27, *p* = 0.05	NS
WASO (min)	F(2, 40) = 0.42, *p* = 0.65	NS
Stage shifts to N1 sleep (nb/h)	F(2, 40) = 5.87, *p* = 0.006	HC < (AUD = KS)
N1 sleep (% of total sleep time)	F(2, 40) = 7.45, *p* = 0.002	HC < (AUD = KS)
N2 sleep (% of total sleep time)	F(2, 40) = 1.59, *p* = 0.21	NS
N3 sleep (% of total sleep time)	F(2, 40) = 5.20, *p* = 0.009	HC > AUD; HC = KS; AUD = KS
REM sleep (% of total sleep time)	F(2, 40) = 5.09, *p* = 0.01	(HC = AUD) > KS

Group effects were tested with parametric ANCOVAs with the quantity of antidepressant (in milligrams) as a covariate, followed by Tukey’s test corrected for unequal sample size. NS: not significant.

**Table A3:** Group effects (HC vs. AUD vs. KS) on objective sleep parameters using ANCOVAs with the quantity of benzodiazepines (in milligrams) as a covariate.

	Statistics	*Post-Hoc* Comparisons
Sleep onset latency	F(2, 40) = 3.69, *p* = 0.03	HC > KS; HC = AUD; AUD = KS
Sleep efficiency %	F(2, 40) = 0.23, *p* = 0.79	NS
Total sleep time (min)	F(2, 40) = 3.03, *p* = 0.06	NS
WASO (min)	F(2, 40) = 1, *p* = 0.37	NS
Stage shifts to N1 sleep (nb/h)	F(2, 40) = 9.36, *p* < 0.001	HC < (AUD = KS)
N1 sleep (% of total sleep time)	F(2, 40) = 8.70, *p* < 0.001	HC < (AUD = KS)
N2 sleep (% of total sleep time)	F(2, 40) = 1.63, *p* = 0.2	NS
N3 sleep (% of total sleep time)	F(2, 40) = 4.52, *p* = 0.01	HC > AUD; HC = KS; AUD = KS
REM sleep (% of total sleep time)	F(2, 40) = 3.54, *p* = 0.03	HC = AUD; HC = KS; KS < AUD

Group effects were tested with parametric ANCOVAs with the quantity of benzodiazepines (equivalent diazepam, in milligrams) as a covariate, followed by Tukey’s test corrected for unequal sample size. NS: not significant.
